# 2,4,6-Tris(1-oxo-2-pyridylsulfanylmeth­yl)mesitylene methanol solvate

**DOI:** 10.1107/S1600536808013081

**Published:** 2008-05-10

**Authors:** B. Ravindran Durai Nayagam, Samuel Robinson Jebas, C. Ravi Samuelraj, Dieter Schollmeyer

**Affiliations:** a Department of Chemistry, Popes College, Sawyerpuram 628 251, Tamil Nadu, India; b Department of Physics, Karunya University, Karunya Nagar, Coimbatore 641 114, India; c Institut für Organische Chemie, Universität Mainz, Duesbergweg 10-14, 55099 Mainz, Germany

## Abstract

In the title compound, C_27_H_27_N_3_O_3_S_3_·CH_4_O, the dihedral angles formed by the mesitylene ring with the three oxopyridyl rings are 89.6 (1), 75.5 (1) and 80.69 (1)°, indicating that all three are nearly perpendicular to the mesitylene ring. Intra­molecular C—H⋯S hydrogen bonds generate *S*(6) ring motifs. The crystal structure is stabilized by intra­molecular C—H⋯S and inter­molecular C—H⋯O hydrogen bonds and weak C—H⋯π inter­actions.

## Related literature

For related literature on the biological activity of *N*-oxides see: Lobana *et al.*, (1989 ▶); Symons & West (1985 ▶); Katsuyuki *et al.* (1991 ▶); Bovin *et al.* (1992 ▶); Leonard *et al.*(1955 ▶). For related literature on *N*-oxides, see: Jebas *et al.* (2005 ▶); Ravindran *et al.* (2008 ▶). For bond-length data, see: Allen *et al.* (1987 ▶); Jebas *et al. *(2005); Ravindran *et al.* (2008 ▶). For hydrogen-bond motifs, see: Bernstein *et al.* (1995 ▶). 
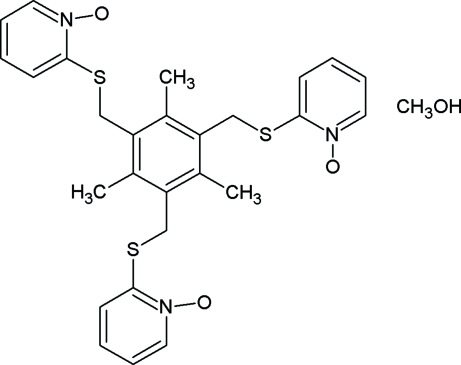


## Experimental

### 

#### Crystal data

C_27_H_27_N_3_O_3_S_3_·CH_4_O
*M* *_r_* = 569.74
Monoclinic, 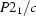

*a* = 11.9644 (17) Å
*b* = 14.9129 (8) Å
*c* = 15.467 (2) Å
β = 91.733 (7)°
*V* = 2758.4 (6) Å^3^
*Z* = 4
Cu *K*α radiation
μ = 2.78 mm^−1^
*T* = 298 (2) K
0.52 × 0.42 × 0.06 mm

#### Data collection

Enraf–Nonius CAD-4 diffractometer
Absorption correction: ψ scan (North *et al.*, 1968 ▶) *T*_min_ = 0.296, *T*_max_ = 0.842
5226 measured reflections
5226 independent reflections
4156 reflections with *I* > 2σ(*I*)
3 standard reflections frequency: 60 min intensity decay: 3%

#### Refinement

*R*[*F* ^2^ > 2σ(*F* ^2^)] = 0.051
*wR*(*F* ^2^) = 0.150
*S* = 1.08
5226 reflections
347 parameters
12 restraints
H-atom parameters constrained
Δρ_max_ = 0.48 e Å^−3^
Δρ_min_ = −0.32 e Å^−3^

### 

Data collection: *CAD-4 Software* (Enraf–Nonius, 1989 ▶); cell refinement: *CAD-4 Software*; data reduction: *CAD-4 Software*; program(s) used to solve structure: *SHELXS97* (Sheldrick, 2008 ▶); program(s) used to refine structure: *SHELXL97* (Sheldrick, 2008 ▶); molecular graphics: *SHELXTL* (Sheldrick, 2008 ▶); software used to prepare material for publication: *SHELXL97*.

## Supplementary Material

Crystal structure: contains datablocks global, I. DOI: 10.1107/S1600536808013081/at2565sup1.cif

Structure factors: contains datablocks I. DOI: 10.1107/S1600536808013081/at2565Isup2.hkl

Additional supplementary materials:  crystallographic information; 3D view; checkCIF report

## Figures and Tables

**Table 1:** Hydrogen-bond geometry (Å, °)

*D*—H⋯*A*	*D*—H	H⋯*A*	*D*⋯*A*	*D*—H⋯*A*
O1*L*—H1*L*⋯O27	0.82	2.41	2.804 (7)	110
C16—H16*A*⋯O36^i^	0.96	2.47	3.348 (4)	152
C16—H16*B*⋯S20	0.96	2.68	3.420 (3)	135
C18—H18*B*⋯S29	0.96	2.79	3.515 (3)	133
C25—H25⋯O7^ii^	0.93	2.44	3.147 (5)	133
C28—H28*A*⋯O7^i^	0.97	2.48	3.397 (3)	157
C31—H31⋯O27^iii^	0.93	2.35	3.107 (4)	139
C2—H2⋯*Cg*1^iv^	0.93	2.91	3.774 (3)	154
C4—H4⋯*Cg*1^v^	0.93	2.67	3.377 (3)	134

Symmetry codes: (i) 
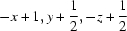
; (ii) 
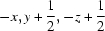
; (iii) 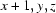; (iv) 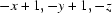; (v) 
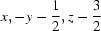
. *Cg*1 is the centroid of the C10–C15 ring.

**Fig. 1.**
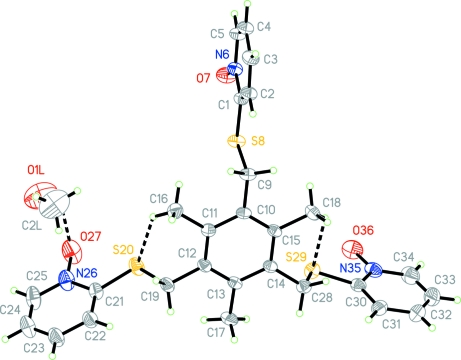
The molecular structure of the title compound, showing 30% probability displacement ellipsoids and the atomic numbering scheme.

## supplementary crystallographic information

### Comment

N-Oxides and their derivatives show a broad spectrum of biological activity, such as antifungal, antibacterial, antimicrobial and antibacterial activities (Lobana & Bhatia, 1989; Symons *et al.*,1985). These compounds are also found to be involved in DNA strand scission under physiological conditions (Katsuyuki *et al.*,1991; Bovin *et al.* 1992). Pyridine N-oxides bearing a sulfur group in position 2 display significant antimicrobial activity (Leonard *et al.*,1955). In view of the importance of N-oxides, we have previously reported the crystal structures of N-oxide derivatives (Jebas *et al.*, 2005; Ravindran *et al.*, 2008). As an extension of our work on these derivatives, we report here the crystal structure of the title compound (Fig. 1).

The bond lengths and angles agree well with the N-oxide derivatives reported earlier (Jebas *et al.*, 2005; Ravindran *et al.*, 2008). The N–O bond length is in good agreement with the mean value of 1.304 (15) Å reported in the literature for pyridine N–oxides (Allen *et al.*,1987).

The meistylene ring is planar with the maximum deviation from planarity being -0.036 (1) Å. The dihedral angle formed by the meistylene ring with the oxopyridinium rings (C1–C5/N6) 89.6 (1) °; (C21–C25/N26) 75.5 (1) ° and (C30–C34/N35) 80.69 (1) ° respectively, indicating that all the three oxopyridinium rings are perpendicular to the meistylene ring.

Intramolecular C—H···S hydrogen bonds generate S(6)S(6) ring motifs. The crystal structure is stabilized by intramolecular C—H···S and intermolecular C—H··· O hydrogen bonds and weak C—H···π interactions (Table 1, where Cg1 is the centroid of the ring C10-C15).

### Experimental

A mixture of tris(bromomethyl)mesitylene (0.399 g, 1 mmol) and 1-hydroxypyridine-2-thione sodium salt (0.448 g, 3 mmol) in water (30 ml) and methanol (30 ml) was heated at 333 K with stirring for 30 min. The compound formed was filtered off, and dried (0.494 g, 92%). The compound was recrystallized from chloroform-methanol (1:2 *v*/*v*).

### Refinement

H atoms were positioned geometrically [C—H = 0.93 (aromatic), 0.96 Å (methyl) 0.97 Å (methylene), and 0.82Å O—H] and refined using a riding model, with *U*_iso_(H) = 1.2 or -1.5*U*_eq_(C).

### Crystal data

**Table:**

C_27_H_27_N_3_O_3_S_3_·CH_4_O	*F*_000_ = 1200
*M**_r_* = 569.74	*D*_x_ = 1.372 Mg m^−^^3^
Monoclinic, *P*2_1_/*c*	Cu *K*α radiation λ = 1.54178 Å
Hall symbol: -P 2ybc	Cell parameters from 25 reflections
*a* = 11.9644 (17) Å	θ = 61–69º
*b* = 14.9129 (8) Å	µ = 2.78 mm^−^^1^
*c* = 15.467 (2) Å	*T* = 298 (2) K
β = 91.733 (7)º	Block, colourless
*V* = 2758.4 (6) Å^3^	0.52 × 0.42 × 0.06 mm
*Z* = 4	

### Data collection

**Table:**

Enraf–Nonius CAD-4 diffractometer	θ_max_ = 69.9º
Monochromator: graphite	θ_min_ = 3.7º
ω/2θ scans	*h* = 0→14
Absorption correction: ψ scan(North *et al.*, 1968)	*k* = −18→0
*T*_min_ = 0.296, *T*_max_ = 0.842	*l* = −18→18
5226 measured reflections	3 standard reflections
5226 independent reflections	every 60 min
4156 reflections with *I* > 2σ(*I*)	intensity decay: 3%
*R*_int_ = 0	

### Refinement

**Table:**

Refinement on *F*^2^	12 restraints
Least-squares matrix: full	H-atom parameters constrained
*R*[*F*^2^ > 2σ(*F*^2^)] = 0.051	*w* = 1/[σ^2^(*F*_o_^2^) + (0.0834*P*)^2^ + 0.7123*P*] where *P* = (*F*_o_^2^ + 2*F*_c_^2^)/3
*wR*(*F*^2^) = 0.150	(Δ/σ)_max_ = 0.001
*S* = 1.09	Δρ_max_ = 0.49 e Å^−^^3^
5226 reflections	Δρ_min_ = −0.32 e Å^−^^3^
347 parameters	Extinction correction: none

### Special details

**Table:**

Geometry. All e.s.d.'s (except the e.s.d. in the dihedral angle between two l.s. planes) are estimated using the full covariance matrix. The cell e.s.d.'s are taken into account individually in the estimation of e.s.d.'s in distances, angles and torsion angles; correlations between e.s.d.'s in cell parameters are only used when they are defined by crystal symmetry. An approximate (isotropic) treatment of cell e.s.d.'s is used for estimating e.s.d.'s involving l.s. planes.

### Fractional atomic coordinates and isotropic or equivalent isotropic displacement parameters (Å^2^)

**Table:**

	*x*	*y*	*z*	*U*_iso_*/*U*_eq_	
C1	0.3585 (2)	0.25409 (17)	−0.03619 (16)	0.0411 (6)	
C2	0.4031 (2)	0.2831 (2)	−0.11233 (17)	0.0509 (7)	
H2	0.4415	0.3372	−0.114	0.061*	
C3	0.3906 (3)	0.2318 (2)	−0.18594 (19)	0.0595 (8)	
H3	0.4189	0.2516	−0.2379	0.071*	
C4	0.3357 (3)	0.1508 (2)	−0.1818 (2)	0.0627 (8)	
H4	0.3283	0.1149	−0.2307	0.075*	
C5	0.2921 (3)	0.1234 (2)	−0.1054 (2)	0.0593 (8)	
H5	0.2552	0.0686	−0.1028	0.071*	
N6	0.30179 (19)	0.17480 (15)	−0.03383 (15)	0.0475 (5)	
O7	0.2579 (2)	0.15045 (15)	0.03880 (14)	0.0660 (6)	
S8	0.36161 (6)	0.30782 (4)	0.06449 (4)	0.04599 (19)	
C9	0.4460 (2)	0.40587 (17)	0.04115 (15)	0.0442 (6)	
H9A	0.5203	0.3879	0.0245	0.053*	
H9B	0.4117	0.4404	−0.0057	0.053*	
C10	0.4525 (2)	0.46111 (16)	0.12348 (15)	0.0380 (5)	
C11	0.3677 (2)	0.52357 (17)	0.13909 (16)	0.0407 (5)	
C12	0.3725 (2)	0.57310 (16)	0.21599 (16)	0.0401 (5)	
C13	0.4580 (2)	0.55854 (16)	0.27863 (16)	0.0401 (5)	
C14	0.5453 (2)	0.49992 (16)	0.25966 (15)	0.0384 (5)	
C15	0.5413 (2)	0.44950 (16)	0.18286 (15)	0.0384 (5)	
C16	0.2719 (3)	0.5372 (2)	0.07464 (19)	0.0551 (7)	
H16A	0.2881	0.5871	0.0378	0.083*	
H16B	0.2047	0.5491	0.1049	0.083*	
H16C	0.2621	0.4841	0.0401	0.083*	
C17	0.4548 (3)	0.6036 (2)	0.36637 (19)	0.0588 (8)	
H17A	0.4966	0.6585	0.3652	0.088*	
H17B	0.487	0.5645	0.4096	0.088*	
H17C	0.3786	0.6164	0.3799	0.088*	
C18	0.6333 (3)	0.3827 (2)	0.16668 (19)	0.0535 (7)	
H18A	0.6067	0.3384	0.126	0.08*	
H18B	0.6552	0.354	0.2201	0.08*	
H18C	0.6964	0.4133	0.1437	0.08*	
C19	0.2853 (2)	0.64413 (18)	0.2303 (2)	0.0489 (6)	
H19A	0.2688	0.6758	0.1766	0.059*	
H19B	0.313	0.6872	0.2728	0.059*	
S20	0.15895 (7)	0.59076 (5)	0.26844 (6)	0.0598 (2)	
C21	0.0714 (2)	0.6832 (2)	0.2755 (2)	0.0534 (7)	
C22	0.0959 (3)	0.7726 (2)	0.2634 (2)	0.0627 (8)	
H22	0.167	0.7891	0.2465	0.075*	
C23	0.0164 (3)	0.8378 (3)	0.2760 (3)	0.0754 (10)	
H23	0.0333	0.898	0.2674	0.09*	
C24	−0.0876 (3)	0.8128 (3)	0.3012 (3)	0.0837 (12)	
H24	−0.1412	0.8561	0.3121	0.1*	
C25	−0.1127 (3)	0.7240 (3)	0.3104 (3)	0.0831 (12)	
H25	−0.1841	0.7072	0.3263	0.1*	
N26	−0.0351 (2)	0.6605 (2)	0.29666 (19)	0.0687 (8)	
O27	−0.0591 (2)	0.57455 (19)	0.3027 (2)	0.0984 (10)	
C28	0.6442 (2)	0.49055 (18)	0.32203 (17)	0.0450 (6)	
H28A	0.6531	0.5448	0.3561	0.054*	
H28B	0.712	0.4813	0.2903	0.054*	
S29	0.62016 (6)	0.39480 (5)	0.39312 (5)	0.0517 (2)	
C30	0.7555 (2)	0.37491 (19)	0.43343 (17)	0.0475 (6)	
C31	0.8492 (3)	0.4281 (2)	0.4247 (2)	0.0604 (8)	
H31	0.8431	0.4844	0.3993	0.073*	
C32	0.9529 (3)	0.3962 (3)	0.4545 (3)	0.0771 (10)	
H32	1.0171	0.4301	0.4475	0.093*	
C33	0.9589 (4)	0.3142 (3)	0.4943 (3)	0.0871 (13)	
H33	1.0278	0.2922	0.5142	0.104*	
C34	0.8648 (4)	0.2647 (3)	0.5050 (2)	0.0772 (11)	
H34	0.8701	0.2094	0.5327	0.093*	
N35	0.7633 (2)	0.29458 (17)	0.47588 (16)	0.0572 (6)	
O36	0.6727 (2)	0.24782 (16)	0.48565 (16)	0.0756 (7)	
O1L	−0.1572 (5)	0.5507 (4)	0.1374 (4)	0.205 (2)	
H1L	−0.12	0.5136	0.1649	0.308*	
C2L	−0.0867 (7)	0.5996 (6)	0.0829 (5)	0.189 (3)	
H2LA	−0.0521	0.6478	0.115	0.284*	
H2LB	−0.1301	0.6237	0.0351	0.284*	
H2LC	−0.03	0.5606	0.0615	0.284*	

### Atomic displacement parameters (Å^2^)

**Table:**

	*U*^11^	*U*^22^	*U*^33^	*U*^12^	*U*^13^	*U*^23^
C1	0.0480 (14)	0.0322 (12)	0.0432 (13)	0.0010 (10)	0.0013 (11)	−0.0040 (10)
C2	0.0599 (16)	0.0485 (16)	0.0447 (14)	−0.0036 (13)	0.0087 (12)	−0.0060 (12)
C3	0.0659 (19)	0.066 (2)	0.0471 (15)	0.0032 (16)	0.0085 (14)	−0.0108 (14)
C4	0.0639 (18)	0.065 (2)	0.0594 (18)	0.0085 (16)	−0.0044 (15)	−0.0287 (16)
C5	0.0639 (18)	0.0428 (16)	0.071 (2)	−0.0009 (14)	−0.0044 (15)	−0.0194 (14)
N6	0.0517 (12)	0.0371 (12)	0.0536 (13)	−0.0005 (10)	0.0007 (10)	−0.0030 (10)
O7	0.0869 (16)	0.0497 (12)	0.0620 (13)	−0.0199 (11)	0.0136 (12)	0.0040 (10)
S8	0.0649 (4)	0.0383 (3)	0.0351 (3)	−0.0115 (3)	0.0081 (3)	−0.0010 (2)
C9	0.0598 (16)	0.0381 (14)	0.0352 (12)	−0.0108 (12)	0.0078 (11)	−0.0017 (10)
C10	0.0494 (14)	0.0304 (12)	0.0346 (12)	−0.0079 (10)	0.0060 (10)	0.0004 (9)
C11	0.0465 (13)	0.0332 (12)	0.0425 (13)	−0.0049 (10)	0.0034 (11)	0.0048 (10)
C12	0.0450 (13)	0.0290 (12)	0.0467 (13)	−0.0001 (10)	0.0082 (11)	0.0028 (10)
C13	0.0497 (14)	0.0294 (12)	0.0416 (13)	0.0001 (10)	0.0079 (11)	−0.0017 (10)
C14	0.0464 (13)	0.0305 (12)	0.0384 (12)	−0.0026 (10)	0.0037 (10)	0.0024 (10)
C15	0.0477 (13)	0.0293 (12)	0.0387 (12)	−0.0008 (10)	0.0094 (10)	0.0007 (9)
C16	0.0574 (17)	0.0541 (17)	0.0534 (16)	0.0011 (14)	−0.0053 (13)	0.0049 (13)
C17	0.074 (2)	0.0518 (17)	0.0513 (16)	0.0057 (15)	0.0062 (14)	−0.0164 (13)
C18	0.0585 (17)	0.0464 (16)	0.0557 (16)	0.0127 (13)	0.0063 (13)	−0.0059 (13)
C19	0.0485 (14)	0.0331 (13)	0.0655 (17)	0.0037 (11)	0.0095 (13)	0.0016 (12)
S20	0.0567 (4)	0.0417 (4)	0.0823 (5)	0.0054 (3)	0.0209 (4)	0.0093 (3)
C21	0.0492 (15)	0.0523 (17)	0.0592 (17)	0.0065 (13)	0.0094 (13)	0.0061 (13)
C22	0.0565 (17)	0.0502 (17)	0.082 (2)	0.0055 (14)	0.0066 (16)	0.0005 (16)
C23	0.073 (2)	0.054 (2)	0.100 (3)	0.0166 (18)	0.006 (2)	0.0046 (19)
C24	0.074 (2)	0.078 (3)	0.099 (3)	0.032 (2)	0.014 (2)	0.010 (2)
C25	0.0563 (19)	0.096 (3)	0.099 (3)	0.020 (2)	0.0250 (19)	0.020 (2)
N26	0.0583 (15)	0.0674 (18)	0.0815 (19)	0.0050 (14)	0.0231 (14)	0.0188 (15)
O27	0.0813 (18)	0.0709 (17)	0.146 (3)	−0.0036 (14)	0.0488 (18)	0.0273 (17)
C28	0.0495 (14)	0.0383 (14)	0.0471 (14)	−0.0038 (11)	−0.0007 (12)	0.0030 (11)
S29	0.0522 (4)	0.0503 (4)	0.0524 (4)	−0.0049 (3)	−0.0013 (3)	0.0111 (3)
C30	0.0598 (16)	0.0425 (14)	0.0399 (13)	0.0037 (12)	−0.0041 (12)	−0.0029 (11)
C31	0.0605 (18)	0.0562 (18)	0.0639 (18)	−0.0008 (15)	−0.0104 (15)	−0.0032 (15)
C32	0.058 (2)	0.085 (3)	0.087 (3)	0.0017 (18)	−0.0141 (18)	−0.015 (2)
C33	0.080 (3)	0.092 (3)	0.087 (3)	0.030 (2)	−0.032 (2)	−0.016 (2)
C34	0.101 (3)	0.066 (2)	0.064 (2)	0.025 (2)	−0.022 (2)	0.0010 (17)
N35	0.0801 (18)	0.0444 (14)	0.0467 (13)	0.0080 (13)	−0.0035 (12)	0.0002 (11)
O36	0.0988 (18)	0.0528 (14)	0.0755 (15)	−0.0065 (13)	0.0083 (14)	0.0178 (12)
O1L	0.213 (4)	0.187 (4)	0.214 (4)	−0.065 (4)	−0.012 (3)	−0.012 (3)
C2L	0.178 (5)	0.210 (5)	0.179 (5)	−0.041 (4)	−0.017 (4)	0.006 (4)

### Geometric parameters (Å, °)

**Table:**

C1—N6	1.364 (3)	C19—S20	1.822 (3)
C1—C2	1.377 (4)	C19—H19A	0.97
C1—S8	1.751 (2)	C19—H19B	0.97
C2—C3	1.376 (4)	S20—C21	1.737 (3)
C2—H2	0.93	C21—N26	1.368 (4)
C3—C4	1.377 (5)	C21—C22	1.378 (4)
C3—H3	0.93	C22—C23	1.379 (4)
C4—C5	1.368 (5)	C22—H22	0.93
C4—H4	0.93	C23—C24	1.368 (5)
C5—N6	1.349 (4)	C23—H23	0.93
C5—H5	0.93	C24—C25	1.366 (6)
N6—O7	1.306 (3)	C24—H24	0.93
S8—C9	1.820 (3)	C25—N26	1.348 (5)
C9—C10	1.517 (3)	C25—H25	0.93
C9—H9A	0.97	N26—O27	1.318 (4)
C9—H9B	0.97	C28—S29	1.830 (3)
C10—C15	1.394 (4)	C28—H28A	0.97
C10—C11	1.404 (4)	C28—H28B	0.97
C11—C12	1.400 (4)	S29—C30	1.742 (3)
C11—C16	1.510 (4)	C30—N35	1.368 (4)
C12—C13	1.404 (4)	C30—C31	1.384 (4)
C12—C19	1.508 (3)	C31—C32	1.394 (5)
C13—C14	1.400 (3)	C31—H31	0.93
C13—C17	1.516 (4)	C32—C33	1.370 (6)
C14—C15	1.406 (3)	C32—H32	0.93
C14—C28	1.510 (3)	C33—C34	1.361 (6)
C15—C18	1.511 (4)	C33—H33	0.93
C16—H16A	0.96	C34—N35	1.358 (4)
C16—H16B	0.96	C34—H34	0.93
C16—H16C	0.96	N35—O36	1.302 (4)
C17—H17A	0.96	O1L—C2L	1.414 (9)
C17—H17B	0.96	O1L—H1L	0.82
C17—H17C	0.96	C2L—H2LA	0.96
C18—H18A	0.96	C2L—H2LB	0.96
C18—H18B	0.96	C2L—H2LC	0.96
C18—H18C	0.96		
			
N6—C1—C2	120.1 (2)	H18A—C18—H18C	109.5
N6—C1—S8	111.72 (18)	H18B—C18—H18C	109.5
C2—C1—S8	128.2 (2)	C12—C19—S20	108.98 (18)
C3—C2—C1	119.8 (3)	C12—C19—H19A	109.9
C3—C2—H2	120.1	S20—C19—H19A	109.9
C1—C2—H2	120.1	C12—C19—H19B	109.9
C2—C3—C4	119.3 (3)	S20—C19—H19B	109.9
C2—C3—H3	120.4	H19A—C19—H19B	108.3
C4—C3—H3	120.4	C21—S20—C19	100.44 (13)
C5—C4—C3	119.8 (3)	N26—C21—C22	118.4 (3)
C5—C4—H4	120.1	N26—C21—S20	112.7 (2)
C3—C4—H4	120.1	C22—C21—S20	128.9 (2)
N6—C5—C4	120.9 (3)	C21—C22—C23	120.8 (3)
N6—C5—H5	119.6	C21—C22—H22	119.6
C4—C5—H5	119.6	C23—C22—H22	119.6
O7—N6—C5	121.4 (2)	C24—C23—C22	119.0 (4)
O7—N6—C1	118.5 (2)	C24—C23—H23	120.5
C5—N6—C1	120.1 (3)	C22—C23—H23	120.5
C1—S8—C9	100.86 (12)	C25—C24—C23	119.9 (3)
C10—C9—S8	106.53 (16)	C25—C24—H24	120
C10—C9—H9A	110.4	C23—C24—H24	120
S8—C9—H9A	110.4	N26—C25—C24	120.7 (3)
C10—C9—H9B	110.4	N26—C25—H25	119.7
S8—C9—H9B	110.4	C24—C25—H25	119.7
H9A—C9—H9B	108.6	O27—N26—C25	121.3 (3)
C15—C10—C11	120.6 (2)	O27—N26—C21	117.7 (3)
C15—C10—C9	120.3 (2)	C25—N26—C21	121.0 (3)
C11—C10—C9	119.1 (2)	C14—C28—S29	108.76 (17)
C12—C11—C10	119.0 (2)	C14—C28—H28A	109.9
C12—C11—C16	120.1 (2)	S29—C28—H28A	109.9
C10—C11—C16	120.9 (2)	C14—C28—H28B	109.9
C11—C12—C13	121.0 (2)	S29—C28—H28B	109.9
C11—C12—C19	119.0 (2)	H28A—C28—H28B	108.3
C13—C12—C19	120.0 (2)	C30—S29—C28	100.76 (13)
C14—C13—C12	119.0 (2)	N35—C30—C31	120.3 (3)
C14—C13—C17	120.3 (2)	N35—C30—S29	111.7 (2)
C12—C13—C17	120.7 (2)	C31—C30—S29	127.9 (2)
C13—C14—C15	120.3 (2)	C30—C31—C32	119.2 (3)
C13—C14—C28	119.9 (2)	C30—C31—H31	120.4
C15—C14—C28	119.8 (2)	C32—C31—H31	120.4
C10—C15—C14	119.8 (2)	C33—C32—C31	119.2 (4)
C10—C15—C18	121.1 (2)	C33—C32—H32	120.4
C14—C15—C18	119.1 (2)	C31—C32—H32	120.4
C11—C16—H16A	109.5	C34—C33—C32	120.4 (4)
C11—C16—H16B	109.5	C34—C33—H33	119.8
H16A—C16—H16B	109.5	C32—C33—H33	119.8
C11—C16—H16C	109.5	N35—C34—C33	121.1 (4)
H16A—C16—H16C	109.5	N35—C34—H34	119.4
H16B—C16—H16C	109.5	C33—C34—H34	119.4
C13—C17—H17A	109.5	O36—N35—C34	121.7 (3)
C13—C17—H17B	109.5	O36—N35—C30	118.7 (3)
H17A—C17—H17B	109.5	C34—N35—C30	119.6 (3)
C13—C17—H17C	109.5	C2L—O1L—H1L	109.5
H17A—C17—H17C	109.5	O1L—C2L—H2LA	109.5
H17B—C17—H17C	109.5	O1L—C2L—H2LB	109.5
C15—C18—H18A	109.5	H2LA—C2L—H2LB	109.5
C15—C18—H18B	109.5	O1L—C2L—H2LC	109.5
H18A—C18—H18B	109.5	H2LA—C2L—H2LC	109.5
C15—C18—H18C	109.5	H2LB—C2L—H2LC	109.5
			
N6—C1—C2—C3	−0.2 (4)	C13—C14—C15—C10	3.0 (3)
S8—C1—C2—C3	−178.9 (2)	C28—C14—C15—C10	−177.1 (2)
C1—C2—C3—C4	−1.6 (5)	C13—C14—C15—C18	−176.4 (2)
C2—C3—C4—C5	1.6 (5)	C28—C14—C15—C18	3.5 (3)
C3—C4—C5—N6	0.2 (5)	C11—C12—C19—S20	81.2 (3)
C4—C5—N6—O7	178.1 (3)	C13—C12—C19—S20	−99.9 (2)
C4—C5—N6—C1	−1.9 (4)	C12—C19—S20—C21	−177.6 (2)
C2—C1—N6—O7	−178.1 (3)	C19—S20—C21—N26	174.9 (2)
S8—C1—N6—O7	0.8 (3)	C19—S20—C21—C22	−5.5 (4)
C2—C1—N6—C5	1.9 (4)	N26—C21—C22—C23	2.8 (5)
S8—C1—N6—C5	−179.1 (2)	S20—C21—C22—C23	−176.7 (3)
N6—C1—S8—C9	177.65 (19)	C21—C22—C23—C24	0.3 (6)
C2—C1—S8—C9	−3.5 (3)	C22—C23—C24—C25	−2.5 (7)
C1—S8—C9—C10	178.14 (19)	C23—C24—C25—N26	1.5 (7)
S8—C9—C10—C15	92.9 (2)	C24—C25—N26—O27	−177.9 (4)
S8—C9—C10—C11	−86.7 (2)	C24—C25—N26—C21	1.8 (6)
C15—C10—C11—C12	−1.1 (3)	C22—C21—N26—O27	175.8 (3)
C9—C10—C11—C12	178.6 (2)	S20—C21—N26—O27	−4.5 (4)
C15—C10—C11—C16	179.3 (2)	C22—C21—N26—C25	−3.9 (5)
C9—C10—C11—C16	−1.0 (3)	S20—C21—N26—C25	175.7 (3)
C10—C11—C12—C13	−2.6 (4)	C13—C14—C28—S29	94.8 (2)
C16—C11—C12—C13	177.0 (2)	C15—C14—C28—S29	−85.0 (2)
C10—C11—C12—C19	176.3 (2)	C14—C28—S29—C30	162.19 (18)
C16—C11—C12—C19	−4.1 (4)	C28—S29—C30—N35	−168.0 (2)
C11—C12—C13—C14	6.3 (4)	C28—S29—C30—C31	10.1 (3)
C19—C12—C13—C14	−172.5 (2)	N35—C30—C31—C32	4.3 (5)
C11—C12—C13—C17	−172.2 (2)	S29—C30—C31—C32	−173.7 (3)
C19—C12—C13—C17	9.0 (4)	C30—C31—C32—C33	−2.1 (5)
C12—C13—C14—C15	−6.5 (3)	C31—C32—C33—C34	−0.3 (6)
C17—C13—C14—C15	172.0 (2)	C32—C33—C34—N35	0.7 (6)
C12—C13—C14—C28	173.6 (2)	C33—C34—N35—O36	180.0 (3)
C17—C13—C14—C28	−7.9 (4)	C33—C34—N35—C30	1.4 (5)
C11—C10—C15—C14	0.8 (3)	C31—C30—N35—O36	177.5 (3)
C9—C10—C15—C14	−178.9 (2)	S29—C30—N35—O36	−4.2 (3)
C11—C10—C15—C18	−179.8 (2)	C31—C30—N35—C34	−3.9 (4)
C9—C10—C15—C18	0.5 (3)	S29—C30—N35—C34	174.3 (2)

### Hydrogen-bond geometry (Å, °)

**Table:**

*D*—H···*A*	*D*—H	H···*A*	*D*···*A*	*D*—H···*A*
O1L—H1L···O27	0.82	2.41	2.804 (7)	110
C16—H16A···O36^i^	0.96	2.47	3.348 (4)	152
C16—H16B···S20	0.96	2.68	3.420 (3)	135
C18—H18B···S29	0.96	2.79	3.515 (3)	133
C25—H25···O7^ii^	0.93	2.44	3.147 (5)	133
C28—H28A···O7^i^	0.97	2.48	3.397 (3)	157
C31—H31···O27^iii^	0.93	2.35	3.107 (4)	139
C2—H2···Cg1^iv^	0.93	2.91	3.774 (3)	154
C4—H4···Cg1^v^	0.93	2.67	3.377 (3)	134

Symmetry codes: (i) −*x*+1, *y*+1/2, −*z*+1/2; (ii) −*x*, *y*+1/2, −*z*+1/2; (iii) *x*+1, *y*, *z*; (iv) −*x*+1, −*y*+1, −*z*; (v) *x*, −*y*−1/2, *z*−3/2.

## References

[bb1] Allen, F. H., Kennard, O., Watson, D. G., Brammer, L., Orpen, A. G. & Taylor, R. (1987). *J. Chem. Soc. Perkin Trans. 2*, pp. S1–S19.

[bb2] Bernstein, J., Davis, R. E., Shimoni, L. & Chang, N. L. (1995). *Angew. Chem. Int. Ed. Engl.* **34**, 1555–1573.

[bb3] Bovin, D. H. R., Crepon, E. & Zard, S. Z. (1992). *Bull. Soc. Chim. Fr.* **129**, 145–150.

[bb4] Enraf–Nonius (1989). *CAD-4 Software* Enraf–Nonius, Delft, The Netherlands.

[bb5] Jebas, S. R., Balasubramanian, T., Ravidurai, B. & Kumaresan, S. (2005). *Acta Cryst.* E**61**, o2677–o2678.

[bb6] Katsuyuki, N., Carter, B. J., Xu, J. & Hetch, S. M. (1991). *J. Am. Chem. Soc.* **113**, 5099–5100.

[bb7] Leonard, F., Barklay, F. A., Brown, E. V., Anderson, F. E. & Green, D. M. (1955). *Antibiot. Chemother.* pp. 261–264. 24543958

[bb8] Lobana, T. S. & Bhatia, P. K. (1989). *J. Sci. Ind. Res.* **48**, 394–401.

[bb9] North, A. C. T., Phillips, D. C. & Mathews, F. S. (1968). *Acta Cryst.* A**24**, 351–359.

[bb10] Ravindran Durai Nayagam, B., Jebas, S. R., Grace, S. & Schollmeyer, D. (2008). *Acta Cryst.* E**64**, o409. 10.1107/S1600536807068766 PMC2960153 21201437

[bb11] Sheldrick, G. M. (2008). *Acta Cryst.* A**64**, 112–122. 10.1107/S0108767307043930 18156677

[bb12] Symons, M. C. R. & West, D.-X. (1985). *J. Chem. Soc. Dalton Trans.* pp. 379–381.

